# Spinal trigeminal neurons demonstrate an increase in responses to dural electrical stimulation in the orofacial formalin test

**DOI:** 10.1007/s10194-011-0404-7

**Published:** 2011-11-25

**Authors:** Alexey Y. Sokolov, Olga A. Lyubashina, Sergey S. Panteleev

**Affiliations:** 1 Valdman Institute of Pharmacology, St. Petersburg Pavlov State Medical University, Lev Tolstoy street 6/8, St. Petersburg, 197022 Russia; 2 Pavlov Institute of Physiology, Russian Academy of Sciences, nab. Makarova 6, St. Petersburg, 199034 Russia

**Keywords:** Formalin test, Trigeminal, Sensitization, Orofacial, Headache

## Abstract

Primary headaches are often associated with pain in the maxillofacial region commonly classified under the term “orofacial pain” (OFP). In turn, long-lasting OFP can trigger and perpetuate headache as an independent entity, which is able to persist after the resolution of the main disorder. A close association between OFP and headache complicates their cause and effect definition and leads to misdiagnosis. The precise mechanisms underlying this phenomenon are poorly understood, partly because of the deficiency of research-related findings. We combined the animal models of OFP and headache—the orofacial formalin test and the model of trigeminovascular nociception—to investigate the neurophysiological mechanisms underlying their comorbidity. In anesthetized rats, the ongoing activity of single convergent neurons in the spinal trigeminal nucleus was recorded in parallel to their responses to the electrical stimulation of the dura mater before and after the injection of formalin into their cutaneous receptive fields. Subcutaneous formalin resulted not only in the biphasic increase in the ongoing activity, but also in an enhancement of neuronal responses to dural electrical stimulation, which had similar time profile. These results demonstrated that under tonic pain in the orofacial region a nociceptive signaling from the dura mater to convergent trigeminal neurons is significantly enhanced apparently because of the development of central sensitization; this may contribute to the comorbidity of OFP and headache.

## Introduction

Primary headaches, especially migraine and tension-type headaches, are often associated with pain in the maxillofacial region commonly classified under the term “orofacial pain” (OFP) [1, 2]. The latter usually accompanies the temporomandibular joint disorders (TMD), masticatory myofascial lesions as well as sinus-related and odontogenic inflammation or tumor [3, 4].

Close relationship between OFP and headache complicates their cause and effect definition and often serves as a reason to classify these pain syndromes together [3]. Indeed, every primary headache, including cluster headache and paroxysmal hemicrania, can occur in the maxillofacial region atypical for them, i.e. felt as OFP, which leads to misdiagnosis and, as a result, to inadequate treatment [4–8]. Migraineurs often complain of a pain in the face, temporomandibular joint noise, tension and tenderness of the masticatory muscles [9]. In addition, patients with primary headaches are more likely than healthy people to demonstrate dysfunctions in the jaw area, which are commonly attributed to TMD [1, 10].

Contrariwise, the orofacial pathology can be accompanied by typical headache that also complicates the diagnostic definition [11–13]. In this case, the headache per se should be considered as a secondary pain, i.e. as a symptom of the main disorder [14, 15]. However, long-lasting OFP can trigger and perpetuate headache as an independent entity, which is able to persist after the primary disease resolution [4]. In such an event, the comorbid state is described, under which the exacerbating relationship between OFP and headache occurs [3, 10, 13]. As reported, more than half of patients seeking treatment for OFP demonstrate symptoms of concomitant headache commonly classified as tension-type headache, chronic daily headache or migraine [3, 16].

The relatively frequent comorbidity of OFP and headache might be explained by the convergence of orofacial and meningeal inputs in the spinal trigeminal nucleus (STN), which is intimately involved in pathophysiology of both pain syndromes [12, 17]. However, the precise mechanisms underlying synergistic relationship between OFP and headache are poorly understood, partly because of the deficiency of research-related findings.

A valid and suitable animal model of persisting pain in the orofacial region is the formalin test [18–20]. A typical behavioral response to orofacial injection of formalin is biphasic, with a short-lasting early phase and a prolonged late one. In neurophysiological studies, the subcutaneous injection of formalin into the orofacial receptive field of innervating the whole craniofacial region convergent STN neurons produced a prolonged biphasic increase in their ongoing activity with a time course similar to that observed in behavioral experiments [19, 21, 22].

The STN neurons are also known to play a prominent role in pathophysiology of headaches by modulating pain transmission from intracranial structures to higher centers of the brain [23–25]. Therefore, the monitoring of STN neuronal responses to electrical, mechanical or chemical stimulation of the dura mater and meningeal vessels is widely used in animal studies of headache [26–32].

In the present work in anesthetized rats, we combined the animal models of OFP and headache (the orofacial formalin test and the trigeminovascular nociception model) to investigate neurophysiological mechanisms underlying comorbidity of these disorders. Namely, we monitored the ongoing activity of convergent neurons in the STN and studied the changes in their responses to electrical stimulation of the dura mater under formalin-induced inflammation of face tissues.

## Methods

All experiments were performed according to the Ethical Guidelines of the International Association for the Study of Pain. The study protocol and experimental design were approved by the Institutional Animal Care and Use Committees of Saint Petersburg State Medical University and Pavlov Institute of Physiology. Thirty adult male Wistar rats (body weight 300–390 g) were used for the study. The animals were housed 2–5 per cage and maintained on a 12-h light/dark schedule with free access to food and water.

### Anesthesia and surgical preparation

Rats were anesthetized with urethane (800 mg/kg, i.p.; ICN Biomedicals, Aurora, OH, USA) and α-chloralose (60 mg/kg, i.p.; MP Biomedicals, Solon, OH, USA). Catheters were placed into the femoral vein for administration of anesthetics and myorelaxants, and into the femoral artery for continuous monitoring of blood pressure. The trachea was intubated and the head of the animal was fixed in a stereotaxic frame. The neck muscles overlying the cisterna magna were separated along the midline and C1 laminectomy was performed. The dura mater was removed to expose the medulla and C1 spinal cord. A longitudinal parietal craniotomy close to the superior sagittal sinus was performed and the stimulating electrodes were placed on the dura mater. The animal was paralysed with pipecuronium bromide (i.v., 1.2 mg/kg initially, maintenance 0.6 mg/kg as required; Gedeon Richter, Budapest, Hungary) and artificially ventilated with room air (75–100 cycles/min, 2–4 ml per cycle) using a small animal ventilator. Rectal temperature was maintained between 37 and 38°C by means of a servocontrolled heating pad. The depth of anesthesia was assessed by monitoring blood pressure responses to noxious stimulation; supplementary anesthetic was administered when necessary to ensure the absence of gross (>20% from the baseline level) blood pressure fluctuations.

### Electrical stimulation of the dura mater

Bipolar stimulating electrodes had resistance of 50 KΩ and consisted of two varnish-insulated silver wires with beads (0.3 mm in diameter) at the end. The electrodes were placed on the dura mater in close proximity to the superior sagittal sinus or visible blood vessels. The dura mater was stimulated with single rectangular pulses of 25–50 V and duration of 0.8 ms delivered by a computer-controlled stimulator. The stimulus intensity was 1.5 times the response threshold.

### Extracellular recordings

Neuronal activity was recorded by varnish-insulated tungsten microelectrodes (Science Products, Hofheim, Germany) with a tip diameter of 5 μm and resistance of 12 MΩ. The electrodes were lowered into the STN at the level of C1 spinal cord in 4-μm steps using a microdrive unit. The signals from the recording electrode were amplified and passed to the analogue input of the computer A/D converter by means of the multifunctional acquisition card. For on-line acquisition, processing and displaying of data, the custom written software was used. To isolate the activity of single units from adjacent cell potentials and noise, three-level amplitude discrimination was used online. The ongoing activity of trigeminal neurons and their responses to the dural electrical stimulation were analyzed as peristimulus time histograms, such that signals gated through the amplitude discrimination were collected in successive bins of 1 ms. For evoked responses, data were collected from 20 recordings (one per 3 s) over 50 ms after each electrical stimulus. For histograms of ongoing activity pseudo stimulation was used, that is, the same software as that for creating histograms of evoked responses was used but electrical stimulation was not actually applied. The histograms had a sweep length of 500 ms and were created automatically from 50 recordings (one per 1 s). All recorded units apart from responses to the dural electrical stimulation were tested for responses to mechanical stimulation of their dural and facial cutaneous receptive fields by von Frey filaments (North Coast Medical, Morgan Hill, CA, USA). Only neurons demonstrating all three kinds of responses were selected for further testing.

### Subcutaneous injection of formalin

Formalin solution was prepared at 5% in saline from a formalin stock (an aqueous solution of 37% formaldehyde) and injected subcutaneously into the center of the neuronal facial mechanoreceptive field in a volume of 15 μl. The onset of the injection was carried out 10 s after the first instant of needle penetration. Formalin was administered in 20 rats. Other ten animals received subcutaneous injection of isotonic saline and were used as control.

### Experimental protocol

Neuronal activity was studied over 150 min after formalin or saline administration. Recordings of ongoing and electrically evoked neuronal activity with simultaneous creation of peristimulus time histograms were performed before (0 min), and in 5, 10, 20, 30, 40, 50, 60, 75, 90, 105, 120, and 135 min after subcutaneous injection. In all experiments, only one unit was tested in each animal. At the end of the experiment, rats were killed by an overdose of urethane (>3 g/kg, i.v.). The recording sites within the spinal cord were marked by an electrolytic lesion through the recording electrode. After routine histological processing of the tissue, lesion sites were examined under a light microscope.

### Statistical analysis

Using peristimulus histograms, neuronal ongoing activity and electrically evoked responses were expressed as a mean number of spikes per second (spikes/s) or a mean number of spikes per stimulus (spikes/stimulus), respectively. Based on the results of the Shapiro–Wilk test of normality, the nonparametric Friedman, Kruskal–Wallis, Wilcoxon signed rank and Mann–Whitney–Wilcoxon tests were used to determine the significance of changes in neuronal activity following subcutaneous formalin or saline. Statistical significance was set at *P* < 0.05. The data are expressed as the mean value ± SEM. The analysis was carried out using Origin 7.5 (OriginLab, Northampton, MA, USA) and GraphPad InStat 3.02 (GraphPad Software, La Jolla, CA, USA) software package.

## Results

### General properties of neurons

**Fig. 1:**
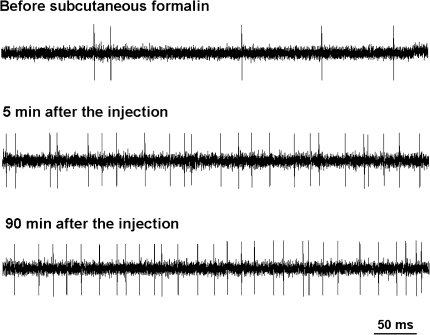
Representative native oscillographic recordings showing changes in the ongoing activity of the spinal trigeminal neuron after subcutaneous injection of formalin

Extracellular recordings were made from 30 neurons within the caudal part of the STN. The recorded neurons were located in the region of the nucleus defined by a rostrocaudal direction from 0.5 to 1.5 mm caudal to the obex and mediolaterally from 2.0 to 2.5 mm left to the middle line at the depth of 0.4–1.2 mm from the dorsal surface of the spinal cord. All of them received convergent afferent inputs from the dura mater and facial skin. Recorded units showed a wide range of frequencies of initial ongoing activity within an interval of 1–22 spikes/s (Fig. 1). The mean rates of ongoing firing in the saline- (*N* = 10) and formalin-treated (*N* = 20) groups did not significantly differ (*P* = 0.48, *U* = 49.0, Mann–Whitney–Wilcoxon test) at 7.3 ± 1.6 and 7.5 ± 1.6 spikes/s, respectively.

**Fig. 2:**
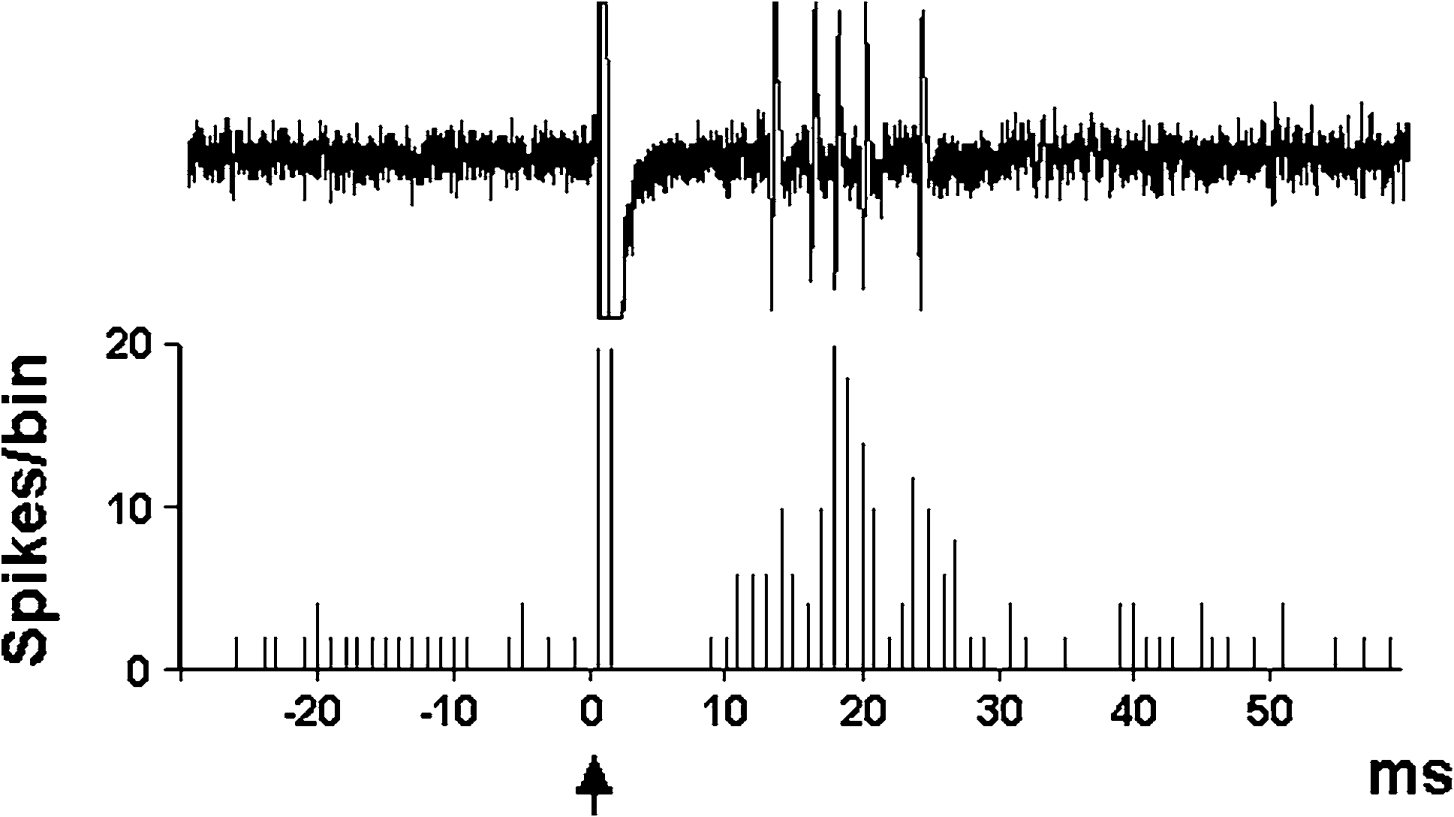
Representative native oscillographic recording and corresponding online produced histogram demonstrating the response of the convergent spinal trigeminal neuron to electrical stimulation of the dura mater. The *arrow* indicates the time of a single electrical stimulus. The histogram is produced from 20 stimuli, bin = 1 ms

The neurons of both experimental groups showed an excitatory response to electrical stimulation of the dura mater with latencies mostly corresponding to the activation of Aδ-fibers (Fig. 2). At baseline, the mean rates of evoked firing were not significantly different between the groups (*P* = 0.82, *U* = 52.0*,* Mann–Whitney–Wilcoxon test). For the saline-treated group, the value was 4.1 ± 0.3 spikes/stimulus *(N* = 10), and for the formalin-treated group, 4.3 ± 0.4 spikes/stimulus *(N* = 20).

All recorded units had facial cutaneous receptive fields and were classified as wide-dynamic range neurons. Their mechanoreceptive fields were located in the periorbital area, on the vibrissa pad, on the upper lip and on the dorsum of the nose.

### Effects of subcutaneous formalin on the ongoing neuronal activity

The injection of saline into the cutaneous receptive field (*N* = 10) did not cause substantial changes in the ongoing neuronal activity. After the administration, the mean rate of ongoing firing in this group was not significantly altered (*P* = 0.14, *Fr* = 6.8, Friedman test) and at each time point was comparable to its baseline level (7.3 ± 1.6 spikes/s, *P* > 0.05, Wilcoxon signed rank test).

**Fig. 3:**
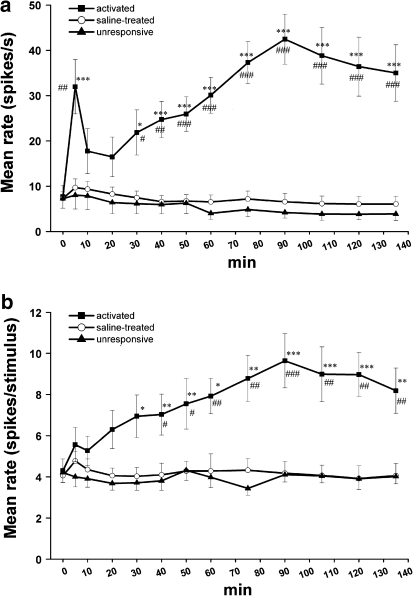
The *line plots* demonstrating the effects of subcutaneous injection of formalin on the ongoing activity of spinal trigeminal neurons (**a**) and their responses to electrical stimulation of the dura mater (**b**). Each *line* represents mean firing rates of neurons in different groups. The data are shown as mean value ± SEM. Significant differences are indicated as follows: ^#^ *P* < 0.05, ^##^ *P* < 0.01, ^###^ *P* < 0.001 versus initial level; *P < 0.05, **P < 0.01, ***P < 0.001 versus saline-treated group

In turn, subcutaneous injection of formalin produced a pronounced increase in ongoing activity in 11 (55%) of formalin-treated units. The Friedman test revealed the maximal level of difference between formalin-induced and baseline firing in this group (*P* < 0.0001, *Fr* = 78.2, Friedman test). The reaction as a rule consisted of two phases. Five minutes after the administration, the neurons showed an increase in the mean discharge rate up to 32.0 ± 6.0 spikes/s (*N* = 11; Fig. 1); this value was significantly higher than the baseline level of ongoing activity of these cells before formalin (7.7 ± 2.5 spikes/s, *N* = 11, *P* = 0.003, Wilcoxon signed rank test) and exceeded firing frequency of saline-treated cells at the same time point (9.7 ± 1.9 spikes/s, *N* = 10, *P* = 0.0008, *U* = 12.0, Mann–Whitney–Wilcoxon test; Fig. 3a). The brief first phase of excitation was followed by a period of relatively low ongoing activity. Twenty minutes after the injection, the mean discharge rate was minimal at 16.5 ± 4.4 spikes/s (*N* = 11); this level was comparable to that prior subcutaneous formalin (*P* = 0.06, Wilcoxon signed rank test; Fig. 3a). The ensuing long-lasting second phase of increased neuronal discharge began 30 min after the injection and persisted until the end of the recording. By 40 min, the mean firing rate of tested neurons increased to 24.7 ± 4.0 spikes/s (*N* = 11); this value was significantly higher than the level prior to formalin administration (*P* = 0.004, Wilcoxon signed rank test) and exceeded the firing frequency of saline-treated units (6.6 ± 1.4 spikes/s, *N* = 10, *P* < 0.0001, *U* = 4.0, Mann–Whitney–Wilcoxon test; Fig. 3a).

The ongoing activity continued to rise and by 90 min reached its maximum at 42.5 ± 5.5 spikes/s (*N* = 11, Figs. 1, 3a). The discharge rate then slightly declined and 135 min after the formalin injection was 35.0 ± 6.2 spikes/s (*N* = 11); this value still significantly exceeded the baseline level (*P* = 0.0002, Wilcoxon signed rank test) and was higher than the firing level of the saline-treated cells at the same time point (6.1 ± 1.7 spikes/s, *N* = 10, *P* = 0.0001, *U* = 7.0, Mann–Whitney–Wilcoxon test). In total, within 50–135 min after the administration the Dunn’s rank sum post hoc analysis for the Friedman test revealed the maximal level of difference between formalin-induced and baseline firing of tested neurons (*P* < 0.001). Between-group comparison showed that the increase in ongoing activity of cells activated by formalin at each point of recording within the same time interval was significant compared to the saline-treated ones (*P* < 0.001, Mann–Whitney–Wilcoxon test; Fig. 3a).

Meanwhile, nine (45%) neurons of the formalin-treated group were unresponsive to the injection of formalin into their cutaneous receptive fields and did not demonstrate any noticeable changes in the ongoing activity. The mean rate of ongoing firing was not significantly altered in this group (*P* = 0.19, *Fr* = 16.1, Friedman test); at each time point after the administration it was comparable to the baseline level (7.3 ± 2.1 spikes/s, *N* = 9, *P* > 0.05, Wilcoxon signed rank test) and did not differ from the discharge rate of the saline-treated cells (*P* > 0.05, Mann–Whitney–Wilcoxon test; Fig. 3a).

Thus, based on the effects of subcutaneous formalin in the ongoing activity, we distinguished two groups of spinal trigeminal neurons—those activating after the injection and unresponsive neurons.

### Effects of subcutaneous formalin on the responses to electrical stimulation of the dura

**Fig. 4:**
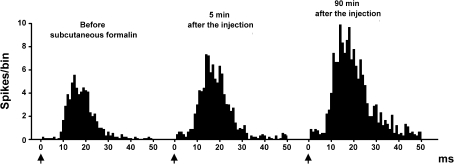
Representative off-line processed histograms showing changes in the response of the spinal trigeminal neuron to dural electrical stimulation after subcutaneous injection of formalin. In each case, the *arrow* indicates the time of a single electrical stimulus. The stimulus artifact is suppressed by the amplitude discrimination. The histograms are produced from 20 stimuli each, bin = 1 ms

A group of neurons demonstrating an increase in ongoing activity after subcutaneous formalin (*N* = 11) showed also significantly enhanced responses to electrical stimulation of the dura mater (*P* < 0.0001, *Fr* = 54.0, Friedman test). The changes in evoked firing had time profile similar to that observed in ongoing activity. Thus 5 min after the administration, the neurons showed an increase in the mean discharge rate up to 5.6 ± 0.8 spikes/stimulus (*N* = 11, Figs. 3b, 4). However, this value did not significantly differ from the baseline level (4.3 ± 0.6 spikes/stimulus, *N* = 11, *P* = 0.28, Wilcoxon signed rank test) and was comparable to electrically induced response of saline-treated cells at the same time point (4.8 ± 0.5 spikes/stimulus, *N* = 10, *P* = 0.45, *U* = 21.0, Mann–Whitney–Wilcoxon test; Fig. 3b). Within the next 5 min, evoked neuronal activity slightly decreased to 5.3 ± 0.7 spikes/stimulus.

Twenty minutes after the formalin administration, the electrically induced firing of tested neurons gradually enhanced and by 40 min, it significantly increased to 7.0 ± 1.0 spikes/stimulus (*N* = 11, *P* = 0.02, Wilcoxon signed rank test). After 90 min, the mean discharge rate was 9.6 ± 1.3 spikes/stimulus (*N* = 11); this value maximally exceeded the baseline level (*P* = 0.0006, Wilcoxon signed rank test; Figs. 3b, 4). Within the period of 105–135 min, the evoked activity fell to 8.2 ± 1.1 spikes/stimulus (*N* = 11); this level was still significantly higher than that prior to formalin administration (*P* = 0.004, Wilcoxon signed rank test). According to the Dunn’s rank sum post hoc analysis for Friedman test, the most significant increase (*P* < 0.001) in electrically induced neuronal activity compared to its baseline level was revealed between 75 and 120 min after subcutaneous formalin. In turn, the difference between evoked firing of formalin- and saline-treated cells became significant 30 min after the injection (*P* = 0.02, *U* = 8.0, Mann–Whitney–Wilcoxon test), was maximal at 90 min (*P* = 0.0003, *U* = 0.0) and slightly declined at the end of recording (*P* = 0.006, *U* = 3.5; Fig. 3b).

In contrast, the group of neurons with formalin-unresponsive ongoing activity (*N* = 9) did not show any noticeable changes in their responses to the electrical stimulation of the dura mater (*P* = 0.19, *Fr* = 15.9, Friedman test). The mean rate of evoked firing of these units at each time point after formalin administration did not differ from the baseline level (4.2 ± 0.5 spikes/stimulus, *N* = 9, *P* > 0.05, Wilcoxon signed rank test) and was comparable to the discharge rate of the saline-treated cells (*P* > 0.05, Mann–Whitney–Wilcoxon test; Fig. 3b).

Thus, trigeminal neurons demonstrating biphasic increase in ongoing activity after subcutaneous formalin were also characterized by the enhancement of responses to electrical stimulation of the dura mater with similar time profile.

## Discussion

This is the first study to demonstrate that subcutaneous injection of formalin into the orofacial receptive field of the STN neurons results not only in biphasic increase in their ongoing activity, but also in an equal enhancement of neuronal responses to electrical stimulation of the dura mater, which has similar time profile. A group of trigeminal neurons, unresponsive to subcutaneous formalin, was also revealed and it did not demonstrate any changes in their ongoing activity or in evoked firing.

The orofacial formalin test is a widely accepted model of tonic pain in the corresponding region, which allows for the study of both behavioral and neurophysiological aspects of this pain condition [18–22]. On the other hand, because the dura mater and major brain vessels innervated by thin unmyelinated trigeminal afferents are known to be the main sources of pain in cephalalgias [23–25], electrical stimulation of the dura is considered to be a valid method of activating the trigeminovascular system and mimicking nociceptive processes occurring during headache [26, 29–32].

Combining the orofacial formalin test and the electrical model of trigeminovascular nociception, we demonstrated that under persisting OFP the nociceptive signaling to STN from the dura mater was significantly facilitated, although the dural afferents were unaffected by experimental inflammation and therefore they were not the primary source of pain. The enhancement of STN neurons responses to electrical stimulation of the dura together with the increase in their ongoing activity indicated that these two processes were inextricably associated. However, unlike changes in the ongoing activity, the significant increase in neuronal responses to the dural stimulation occurred only in the second phase of the formalin test, whereas their alterations in the first phase were comparable to those observed in the saline-treated group. This needs further explanation.

As revealed previously, the expression of the second phase of the formalin test depends primarily on nociceptive signaling from the periphery, i.e. from the tissue affected by formalin-induced inflammation [33, 34]. The central neuroplasticity, if it occurs, seems to play a secondary and therefore less prominent role in the process. In turn, the long-term repetitive electrical stimulation of the dura mater does not produce significant changes in the evoked activity of the STN neurons [35] and it also does not result in their wind-up [36]. Meanwhile, it has been recently shown that in the state of hyperexcitation induced by inflammatory challenge of the dura mater, the spinal trigeminal neurons together with the increase in ongoing activity and reduction of thresholds to mechanical stimulation of the facial receptive field demonstrated significant enhancement of responses to the dural electrical stimulation [30, 31]; this allowed us to consider the latter as an additional marker of central sensitization.

Taking into account everything mentioned above, we suppose that the increase in responses of the STN cells to dural electrical stimulation, observed in the second phase of the orofacial formalin test, can be a result of changes in neuronal excitation, i.e. manifest the development of central sensitization. It is reasonable to suggest that this process is a direct consequence of tonic nociceptive signaling from the peripheral site of formalin-induced inflammation. As evidenced by similar time-courses of changes in ongoing and electrically induced neuronal activities, a persistent nociceptive flow from the periphery not only initiates the sensitization of central trigeminal neurons, but also maintains it. However, the enhanced responsiveness of the STN neurons in the second phase of the formalin test also seemed to contain an autonomous component, independent of peripheral input. This assumption can be supported by the following considerations.

Firstly, after subcutaneous injection of formalin, the STN neurons recorded in the present study demonstrated the facilitation of responses to electrical stimulation of the chemically intact dura mater, which progressed in the course of the experiment. Undoubtedly, it is usually difficult to differentiate between the enhanced excitation of the STN neurons per se and the increased afferent input from the periphery. Indeed, the escalation of ongoing activity, observed in the second phase of the formalin test, can be explained by continuous signaling from the inflammation site. However, taking into account that dural afferents, unlike cutaneous ones, were unaffected by formalin, it is reasonable to suppose that the increase in neuronal responses to the dural electrical stimulation demonstrated within the same time interval was ingenuously conditioned by alterations in the functional state of the STN cells. The significant changes in electrically induced activity occurred only 30 min after subcutaneous injection of formalin, i.e. within the time period, minimally sufficient for the induction of central sensitization [27]. The obtained data are consistent with the results of other experiments on sensitization of spinal trigeminal neurons induced by the topical application of inflammatory soup (IS) on the dura mater [27, 28]. In these experiments, the decrease in thresholds of the STN neurons to the mechanical stimulation of the dura mater, as a result of the local activation of dural nociceptors by IS and subsequent peripheral sensitization, was accompanied by increased sensitivity and expansion of chemically intact neuronal cutaneous receptive fields, which are considered to be an obvious sign of central sensitization.

Secondly, in the present study, the hyperactivity of the STN neurons was observed even 2 h after the onset of the second phase of the formalin test indicating the persistence of this state that is typical for the phenomenon of central sensitization [27]. It should be noted here that the completion of each experiment was not caused by the attenuation of neuronal activity, but was limited by the experimental design. At the same time, the duration of behavioral response in the second phase of the orofacial formalin test does not exceed 30–35 min, which indicates the termination of formalin action and the attenuation of pain signaling from the periphery by that time [18, 22].

As it is known, the STN neurons are characterized by the reception of convergent somatovisceral afferent inputs from both extra- and intracranial tissues. This peculiarity determines the location and irradiation of pain in various OFP and headache and underlies their comorbidity [2, 12, 37, 38]. Our study demonstrates that persisting pain in the orofacial region can promote a significant facilitation of nociceptive transmission from craniovascular structures to the CNS apparently because of the sensitization of the STN convergent neurons, whose activation is considered to be an important component of headache neurobiology. It is generally assumed that a persistent increase in the excitation of these neurons determines the typical clinical manifestations of primary headaches, for example, the occurrence of cutaneous allodynia, and forms the basis of the mechanisms leading to the development of chronic conditions [27, 28, 39]. We suppose that the data obtained in the present study can contribute to understanding of neurophysiological processes underlying the comorbidity of OFP and headache.
